# MicroRNA-221 Induces Cell Survival and Cisplatin Resistance through PI3K/Akt Pathway in Human Osteosarcoma

**DOI:** 10.1371/journal.pone.0053906

**Published:** 2013-01-23

**Authors:** Guangyi Zhao, Chengkui Cai, Tongtao Yang, Xiuchun Qiu, Bo Liao, Wei Li, Zhenwei Ji, Jian Zhao, Haien Zhao, Mingjun Guo, Qiong Ma, Chun Xiao, Qingyu Fan, Baoan Ma

**Affiliations:** Department of Orthopedic Surgery, Orthopedics Oncology Institute of Chinese PLA, Tangdu Hospital, Fourth Military Medical University, Xi'an, Shaanxi, People's Republic of China; H.Lee Moffitt Cancer Center & Research Institute, United States of America

## Abstract

**Background:**

MicroRNAs are short regulatory RNAs that negatively modulate protein expression at a post-transcriptional and/or translational level and are deeply involved in the pathogenesis of several types of cancers. Specifically, microRNA-221 (miR-221) is overexpressed in many human cancers, wherein accumulating evidence indicates that it functions as an oncogene. However, the function of miR-221 in human osteosarcoma has not been totally elucidated. In the present study, the effects of miR-221 on osteosarcoma and the possible mechanism by which miR-221 affected the survival, apoptosis, and cisplatin resistance of osteosarcoma were investigated.

**Methodology/Principal Findings:**

Real-time quantitative PCR analysis revealed miR-221 was significantly upregulated in osteosarcoma cell lines than in osteoblasts. Both human osteosarcoma cell lines SOSP-9607 and MG63 were transfected with miR-221 mimic or inhibitor to regulate miR-221 expression. The effects of miR-221 were then assessed by cell viability, cell cycle analysis, apoptosis assay, and cisplatin resistance assay. In both cells, upregulation of miR-221 induced cell survival and cisplatin resistance and reduced cell apoptosis. In addition, knockdown of miR-221 inhibited cell growth and cisplatin resistance and induced cell apoptosis. Potential target genes of miR-221 were predicted using bioinformatics. Moreover, luciferase reporter assay and western blot confirmed that PTEN was a direct target of miR-221. Furthermore, introduction of PTEN cDNA lacking 3′-UTR or PI3K inhibitor LY294002 abrogated miR-221-induced cisplatin resistance. Finally, both miR-221 and PTEN expression levels in osteosarcoma samples were examined by using real-time quantitative PCR and immunohistochemistry. High miR-221 expression level and inverse correlation between miR-221 and PTEN levels were revealed in osteosarcoma tissues.

**Conclusions/Significance:**

These results for the first time demonstrate that upregulation of miR-221 induces the malignant phenotype of human osteosarcoma whereas knockdown of miR-221 reverses this phenotype, suggesting that miR-221 could be a potential target for osteosarcoma treatment.

## Introduction

Osteosarcoma is the most primary bone tumor and occurs predominantly in adolescents and young adults [1]. Advances in osteosarcoma therapy over the past several decades have enhanced patient outcomes, with most effective regimens currently including neoadjuvant and adjuvant chemotherapy coupled with local control that usually consists of limb-sparing surgery [2]. However, outcome remains poor for most patients with metastatic or recurrent osteosarcoma. The frequent acquisition of drug-resistant phenotypes and the occurrence of second malignancies often associated with chemotherapy remain serious problems. Therefore, the identification of the effector molecules and/or signal pathways responsible for regulating chemotherapy resistant and malignant development is crucial for improving the osteosarcoma treatment level.

MicroRNAs (miRNAs) are a class of 22–25 nucleotide RNA molecules that negatively regulate gene expression in animals and plants [3], [4]. Though miRNAs were first discovered to have crucial functions in Caenorhabditis elegans development [5], recent progress in cancer biology has shown that miRNAs are frequently dysregulated in diverse cancer subtypes including synovial sarcoma, colon cancer [6], breast cancer [7], gliomas [8], glioblastoma [9], hepatocellular carcinoma [10], lung cancer [11] and gastric cancer [12], [13]. It has been proposed that depending on the role of the mRNA targets, miRNAs can function either as tumor suppressors or as oncogenes [14]. miR-221 is clustered on the X chromosome and it has been reported to be overexpressed in many cancers including breast cancer [15], gastric carcinoma [16], melanoma [17], hepatocellular carcinoma (HCC) [18], glioblastoma [19], [20], and prostate carcinoma [21]. miR-221 has been shown as an oncogene in these cancers. However, what function miR-221 exerts in osteosarcoma cells has not been identified.

The PI3K/Akt pathway is well known to be a major cell survival pathway in many cancers [22]–[25] including osteosarcoma [26]–[29]. As a key molecule of this pathway, Akt regulates several downstream targets including the apoptosis-inducing protein CCND1 [30], p27 [31], BAD [32], resulting in cell growth, survival and cisplatin resistance. As one of the targets of phoshoinositide3-kinase (PI3K) [33], Akt contains the pleckstrin homology domain which directly binds phosphatidylinositol-3,4,5-trisphosphate (PIP3), a product of PI3K activation. Akt activity depends heavily on the availability of PIP3, phosphatases such as PTEN and SHIP [34] act as potent negative regulators of its activity. PTEN expression is considered to be an important negative regulator controlling the PI3K/Akt activation [35]. This gene is an important regulator of protein phosphatases and 3′-phosphoinositol phosphatases. PTEN dephosphorylates phosphatidylinositol-3,4,5-triphosphate (PIP3), the second messenger produced by phosphoinositide 3-kinase (PI3K), to negatively regulate the activity of the serine/threonine protein kinase, Akt [31], [34].

In this report, we demonstrated that miR-221 induced cell proliferation, inhibited cell apoptosis and enhanced cisplatin resistance in both human osteosarcoma cell lines SOSP-9607 and MG63. In addition, we showed that miR-221 negatively regulated PTEN by binding to its 3′-UTR leading to inhibition of PTEN translation and activation of Akt pathway. Moreover, several downstream genes of pAkt, such as BCL-2 and CCND1, p27 were regulated by miR-221. Furthermore, restoring expression of PTEN or PI3K/AKT inhibitor LY294002 recovered the cisplatin sensitivity in the both cells. Finally, we observed miR-221 was upregulated in human osteosarcoma samples. These findings indicate that miR-221 induce cell survival and cisplatin resistance in human osteosarcoma at least partly through targeting the PI3K/PTEN/Akt pathway. We supposed that miR-221 could be a promising gene therapeutic agent in osteosarcoma with its oncogene role through down-regulating multiple tumor suppressor targets.

## Results

### Upregulation of miR-221 and validation for miR-221 oligonucleotides transfection in human osteosarcoma cell lines

**Figure 1:**
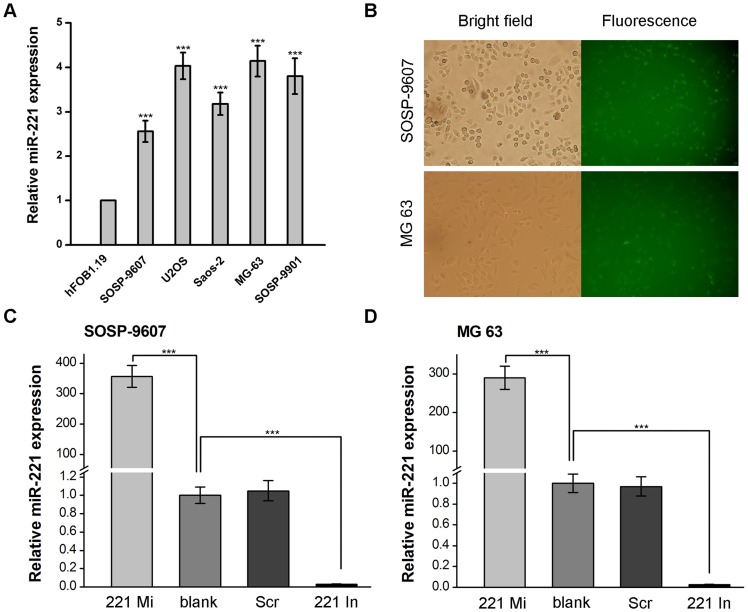
miR-221 expression and miR-221 oligonucleotides transfection in osteosarcoma cells. **A**, the expression of miR-221 was analyzed by qRT-PCR and normalized to RNU24 in five osteosarcoma cell lines. The expression of miR-221 in hFOB1.19 osteoblasts was used as a control. For **B**–**D**, SOSP-9607 and MG 63 cells were transfected with miR-221 mimic (221 Mi), miR-221 inhibitor (221 In), or scramble oligonucleotides (Scr). The blank (not transfected) cells were used as control. 24 h after transfection, the cells were observed and miR-221 expression levels were evaluated by qRT-PCR analysis. **B**, SOSP-9607 cells (**top**) and MG 63 cells (**bottom**) were observed with white bright (**left**) and green fluorescence assay (**right**) in the same vision using fluorescence microscopy (200×). **C**, miR-221 expression levels were evaluated by qRT-PCR analysis in transfected SOSP-9607 cells. **D**, miR-221 expression levels were assayed in transfected MG63 cells. RNU24 was used as an internal loading control to normalize the results. For **C** and **D**, data was presented as means ± SD. Columns, mean of three independent experiments; bars, SD; ***, p<0.001.

To elucidate the role of miR-221 in human osteosarcoma, we evaluated the expression levels of miR-221 in five human osteosarcoma cell lines, SOSP-9607, U2OS, Saos-2, MG63 and SOSP-9901, as well as hFOB1.19 osteoblasts by using real-time quantitative PCR. The hFOB1.19 osteoblasts was used as control. Although MG63 had the highest one and SOSP-9607 had the lowest one, the expression levels of miR-221 in investigated osteosarcoma cell lines were all significantly higher compared with that of hFOB1.19 osteoblasts cells. (each fold change >2.5, p<0.001) (Figure 1A). Thus, these results indicate that miR-221 is significantly upregulated in osteosarcoma cell lines.

To investigate the function of miR-221, we transfected human osteosarcoma cell lines SOSP-9607 (with the lowest miR-221 expression level in the five osteosarcoma cell lines) and MG63 (had the highest expression level of miR-221) with 100 nM chemically synthesized double-stranded oligonucleotides (miR-221 mimic) that mimic the endogenous mature miR-221 function, 100 nM modified antisense oligonucleotides that inhibit miR-221 function (miR-221 inhibitor) or 100 nM scramble oligonucleotides. The cells transfected with nothing (blank) were used as control. These oligonucleotides could be observed with fluorescence microscopy (OLYMPUS) because there was a FAM fluorescent label in their 5′ oligonucleotide structure. 24 hours after transfection, transfection efficiency (more than 80%) of miR-221 mimic in these two cells were observed by fluorescence microscopy analysis (Figure 1B). miR-221 inhibitor and scramble group had the similar transfection efficiency in these two cells (date not shown).

Additionally, 24 hours after transfection, miR-221 expression were evaluated by real-time quantitative PCR. The results showed that miR-221 mimic increased the expression of miR-221 by about 360-fold (p<0.001) while miR-221 inhibitor decreased the expression was about 65-fold in SOSP-9607 cells (p<0.001) compared with the blank group. However, no statistical significance of miR-221 expression was found between the scramble and blank control groups (Figure 1C). Similar experiments were performed in MG63 cells. The expression of miR-221 was increased by about 290-fold (p<0.001) in miR-221 mimic group while the expression of miR-221 was decreased by about 45-fold (p<0.001) in miR-221 inhibitor group compared with the blank group (Figure 1D). These results indicate that miR-221 mimic and miR-221 inhibitor could regulate miR-221 expression effectively in both SOSP-9607 and MG63 cells. These strategies were then used as the basis of the remaining experiments.

### miR-221 induces cell proliferation in both SOSP-9607 and MG 63 cell lines

**Figure 2:**
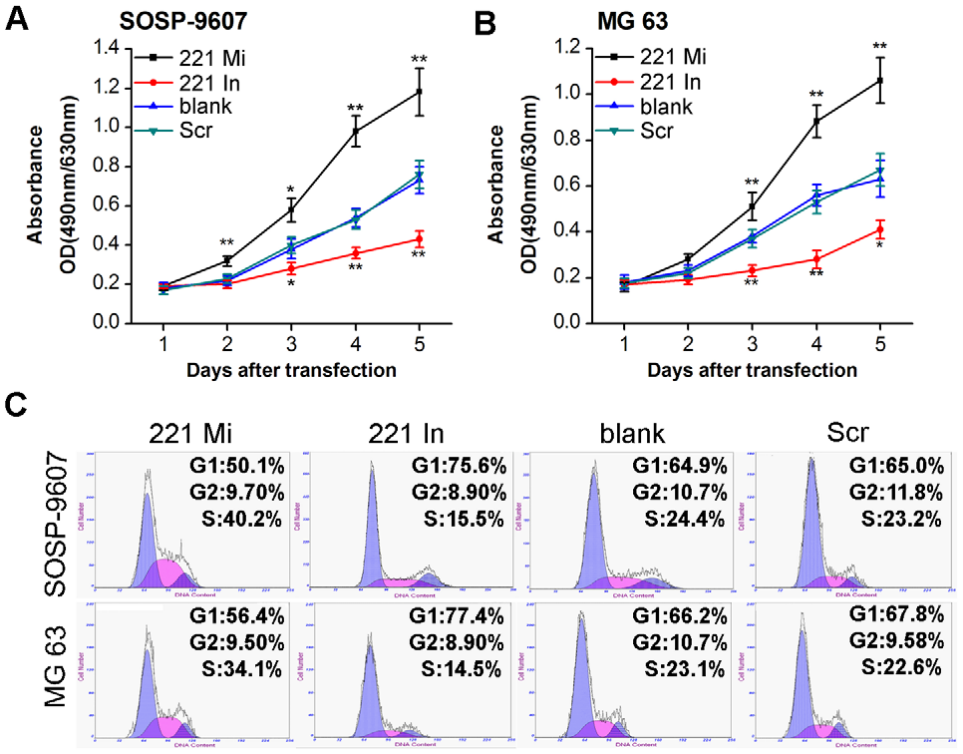
miR-221 induces cell proliferation in both SOSP-9607 and MG63 cell lines. Both SOSP-9607 and MG 63 cells were transfected with miR-221 mimic (221 Mi), miR-221 inhibitor (221 In) or scramble oligonucleotide (Scr). The non-transfected cells (blank) were used as control. After transfection, every 24 h, MTT assay was performed on four groups of SOSP-9607 (**A**) and MG 63 cells (**B**). The viable cell number was evaluated as the value of the absorbance at 490 nm with a reference wavelength of 630 nm. Values of optical density (OD) are expressed as means ± SD. Point symbol, mean of three independent experiments; bars, SD; *, p<0.05, **p<0.01. **C**, 48 h after transfection, cell cycle distribution of treated SOSP-9607 (**top**) and MG 63 (**bottom**) was measured using flow cytometry analysis.

The proliferation rates of SOSP-9607 and MG63 cells with enhanced or silenced expression of miR-221 was determined via MTT assay. MTT assay was performed following the procedure described in Methods every 24 hour. And then proliferation curve was depicted. The results demonstrated that both miR-221 inhibitor transfected cells proliferate at a significantly lower rate as compared with blank cells, respectively. In contrast, both cells with overexpression of miR-221 by infected with miR-221 mimic significantly enhance proliferation as compared with blank cells, respectively. No statistical significance was observed in the proliferation rate between the blank and scramble group, respectively (Figure 2A and B). Cell cycle distribution was detected by flow cytometry. Before transfection, 1×10^5^ cells of SOSP-9607 or MG63 were seeded into each well of six-well plates and incubated overnight. The following day, these cells were transfected with 100 nM miR-221 mimic, miR-221 inhibitor or scramble oligonucleotide, respectively. The blank groups were used as control. Forty eight hours after transfection, cell cycle distribution were measured by flow cytometry. The results showed that the percentage of SOSP-9607 cells treated with the miR-221 mimic and miR-221 inhibitor in the G0/G1 phase was 50.1±2.6% and 75.6±3.2%, respectively, while the blank and scramble group was 64.9±1.9% and 65±2.1%, respectively. The S phase fraction in miR-221 mimic, miR-221 inhibitor, blank and scramble groups were 40.2±3.2%, 15.5±1.2%, 24.4±1.5% and 23.2±2.0%, respectively (Figure 2C, top). Similar experiments were performed in MG63 cells with similar results obtained. The percentage of miR-221 mimic, miR-221 inhibitor, blank control and scramble groups in the G0/G1 phase were 56.4±3.2%, 77.4±3.8%, 66.2±2.9% and 67.8±3.5%, respectively. The S phase fraction in miR-221 mimic, miR-221 inhibitor, blank and scramble groups were 34.1±2.6%, 14.5±1.9%, 23.1±2.3% and 22.6±2.1%, respectively (Figure 2C, bottom). In sum, both in SOSP-9607 and MG63 cells, transfection with miR-221 mimic resulted in the lowest percentage of cells in G0/G1 phase (p = 0.001 for SOSP-9607 cells and p = 0.017 for MG63) but highest fraction in S phase (p = 0.001 for SOSP-9607 cells and p = 0.005 for MG63 cells). In contrast, both SOSP-9607 and MG63 cells treated with miR-221 inhibitor had the higher percentage of cells in G0/G1 phase (p = 0.009 for SOSP-9607 and p = 0.032 for MG63) but lower fraction in S phase (p = 0.005 for SOSP-9607 and p = 0.008 for MG63) as compared with the blank group, respectively. There was no statistical significance between blank and scramble control cells in the G0/G1 phase (p = 0.954 for SOSP-9607 and p = 0.575 for MG63) and S phase (p = 0.453 for SOSP-9607 and p = 0.795 for MG63). No statistical significance was observed in the percentage of cells in the G2/M phase among the four groups in this two cell lines. These data demonstrate that miR-221 may induce the proliferation in both SOSP-9607 and MG 63 cells.

### miR-221 regulates apoptosis in both SOSP-9607 and MG63 cells

**Figure 3:**
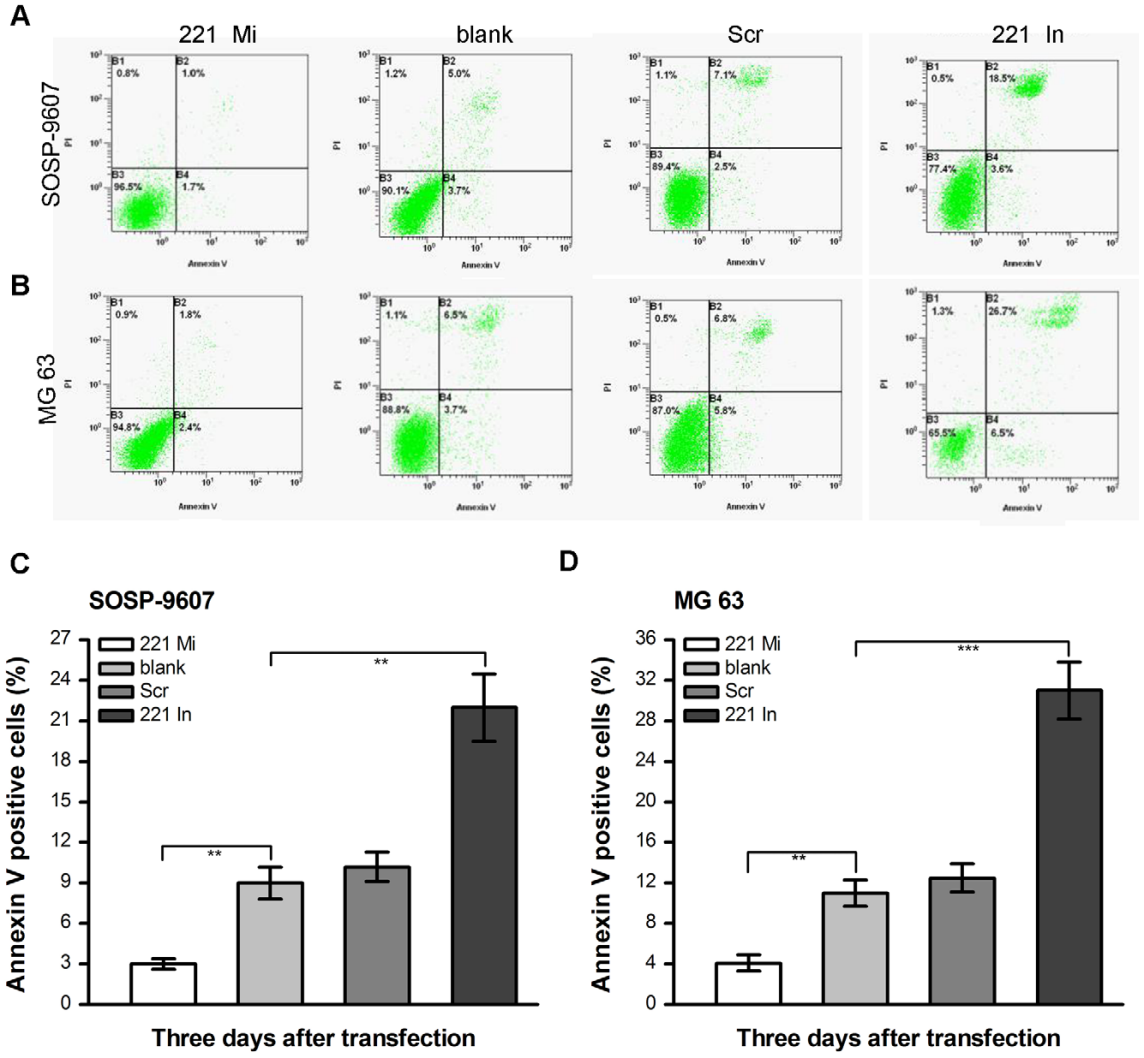
miR-221 regulates apoptosis in both SOSP-9607 and MG63 cells. **A, C** SOSP-9607 cells were transfected with 100 nM miR-221 mimic (221 Mi), miR-221 inhibitor (221 In) or scramble oligonucleotide (Scr) for 48 h. The blank cells were used as control. Apoptosis was measured by FACS with Annexin V and propidium iodide staining. **B, D** Apoptosis of MG 63 was measured by FACS with Annexin V and propidium iodide staining. The data were presented as the means ± SD in panel **C** and panel **D**. Columns, mean of three independent experiments; bars, SD; **, p<0.01, ***, p<0.001.

We next investigated whether miR-221 could regulate apoptosis in SOSP-9607 cell line. The day before transfection, SOSP-9607 cells were seeded into six-well plates at a density of 1×10^5^ cells/well. The following day, cells were transfected with 100 nM miR-221 mimic, miR-221 inhibitor, or scramble oligonucleotide, respectively. The blank groups was used as control. 48 h after transfection, apoptosis was detected by annexin-V/propidiumiodide-double staining-based flow cytometry analysis. The results showed that SOSP-9607 cells transfected with miR-221 mimic significantly decreased spontaneous apoptosis as compared with the blank group (p = 0.008). However, SOSP-9607 cells transfected with miR-221 inhibitor significantly promoted spontaneous apoptosis as compared with blank control group (p = 0.001). The cells transfected with scramble did not produce noticeable changes as compared to blank control (p = 0.236) (Figure 3A and C). The similar results were obtained in MG63 cell lines. Apoptosis was much lower in MG63 cells transfected with miR-221 mimic than in blank group cells (p = 0.001) and much higher in cells transfected with miR-221 inhibitor than the blank group cells (p<0.001). The cells transfected with scramble oligonucleotide and blank cells had similar apoptosis rate (p = 0.524) (Figure 3B and D).

### Involvement of miR-221 in cisplatin resistance in both SOSP-9607 and MG63 cells

**Figure 4:**
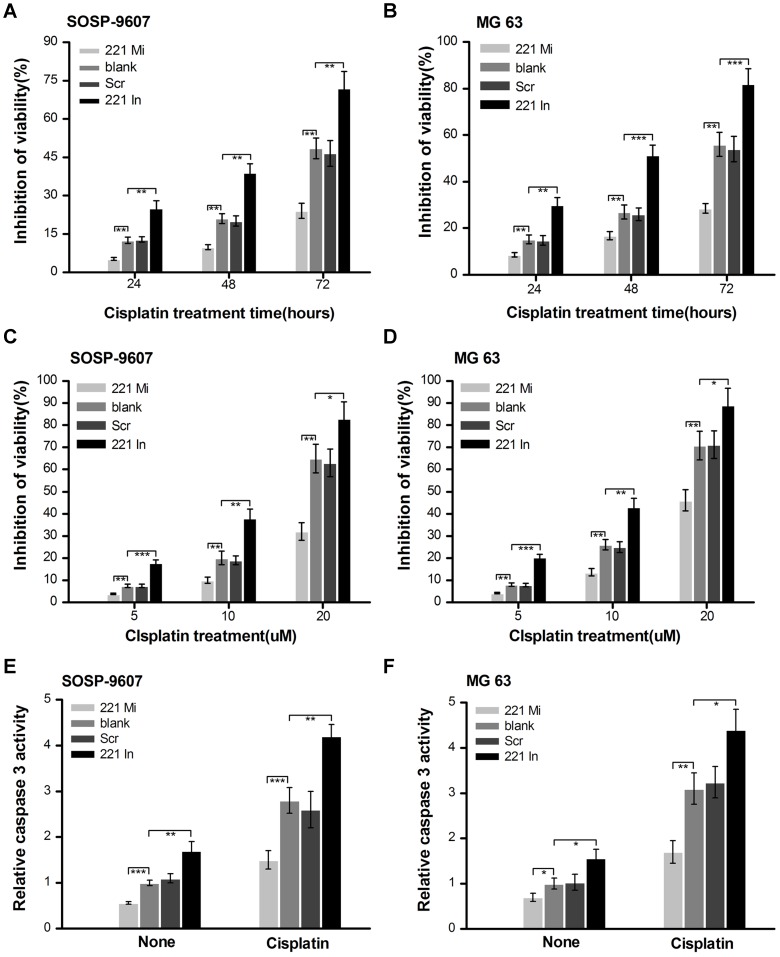
Involvement of miR-221 in cisplatin resistance in both SOSP-9607 and MG63 cells. Cells were transfected with miR-221 mimic (221 Mi), miR-221 inhibitor (221 In) or scramble oligonucleotide (Scr). The blank cells were used as control. Twenty four hours after transfection, treated cells were seeded into 96-well plates at the density of 5×10^3^ per well. **A** and **B**, twenty four hours later, SOSP-9607 and MG 63 cells were treated with or without 10 uM cisplatin for 24 h, 48 h or 72 h. Viability evaluation were examined using MTT assay. Then viability inhibition was calculated. **C** and **D**, twenty four hours later, SOSP-9607 and MG 63 cells were treated with or without the indicated concentrations of cisplatin for 48 h. Then, viability evaluation were examined using MTT assay. **E** and **F**, twenty four hours later, transfected SOSP-9607 and MG 63 cells were treated with or without 10 uM cisplatin for 24 h. Caspase3 activity was measured using Caspase-Glo® 3/7 assay kit (Promega). The data were presented as the means ± SD. Columns, mean of three independent experiments; bars, SD. **, p<0.01, ***, p<0.001.

Cisplatin is known to exert its cancer cell killing effect through induction of apoptosis. Next, we examined whether miR-221 was capable of inhibiting cisplatin-induced apoptosis. SOSP-9607 cells were transfected as described above. Twenty four hours after transfection, treated cells were seeded into 96-well plates at the density of 5×10^3^ per well. Twenty four hours later cells were treated with 10 uM cisplatin or not for 24 h, 48 h or 72 h. MTT assay showed that compared with blank group, overexpression of miR-221 markedly inhibited cisplatin-induced cell cytotoxicity in SOSP-9607 cells (Figure 4A). To confirm and extend our findings, another osteosarcoma cell line MG63, was used to investigate the effect of miR-221 on cisplatin resistance (Figure 4B). Moreover, overexpression of miR-221 decreased cisplatin-induced cytotoxicity and increased cisplatin resistance under various concentrations of cisplatin treatment in both SOSP-9607 and MG63 cells (Figure 4C and D).

Although overexpression of miR-221 conferred cisplatin resistance to cells, we asked whether down-regulation of miR-221 may increase cisplatin sensitivity. miR-221 inhibitor was also transfected into SOSP-9607 cells following treatment with 10 uM cisplatin or not. We found that the knockdown of miR-221 by miR-221 inhibitor increased cisplatin-induced cytotoxicity in SOSP-9607 cells (Figure 4A). Similar experiments were performed in MG-63 cells with similar results obtained (Figure 4B). And more, downexpression of miR-221 increased cisplatin-induced cytotoxicity and decreased cisplatin resistance under various concentrations of cisplatin treatment in both SOSP-9607 (Figure 4C) and MG63 cells (Figure 4D).

Caspases are important regulators of apoptosis. Therefore, the activity of caspase3 was further detected using the Caspase-Glo®3/7 assay system (Promega) in transfected SOSP-9607 cells. These cells were treated with 10 uM cisplatin treatment or not and then activity of caspase3 was detected after treatment for 24 hours. Lower caspase3 activity was found in miR-221 mimic transfected cells compared with blank control groups while higher caspase3 activity was found in miR-221 inhibitor transfected cells compared with the blank control groups. No statistical significance of caspase3 activity was observed between the blank control and scramble control groups (Figure 4E). Similar results were obtained in the MG63 cells (Figure 4F). Taken collectively, these data indicate that miR-221 could play an important role in cisplatin resistance in osteosarcoma cells.

### miR-221 targets PTEN leading to activation of the Akt pathway

**Figure 5:**
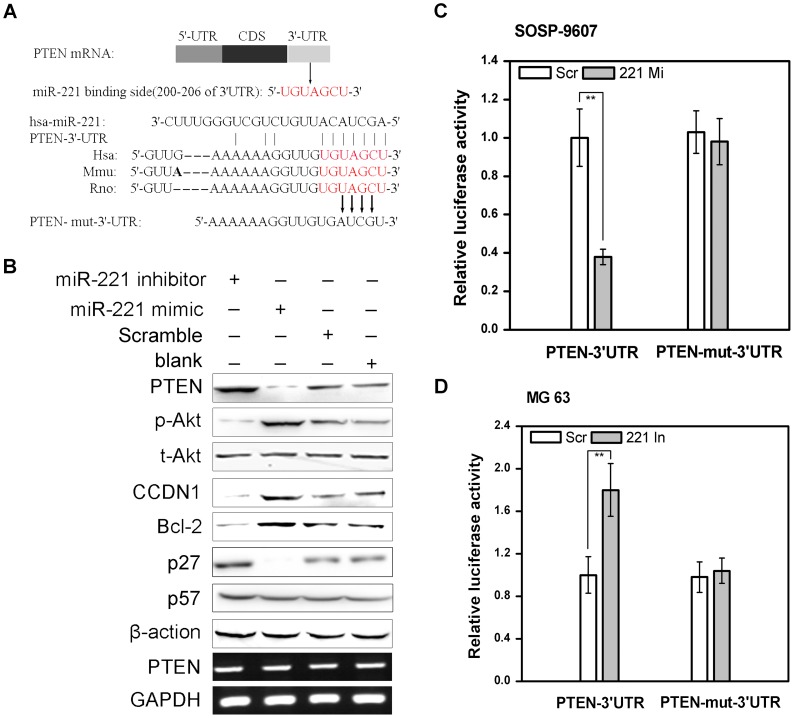
miR-221 targets PTEN leading to activation of the Akt pathway. **A**, putative miR-221-binding sites (red) in the 3′-UTRs of PTEN (**top**). The **middle** panel shows sequence alignment of the miR-221 sequences (red color) with a region of the PTEN 3′-UTR (red) from indicated species. The **bottom** shows a mutant of PTEN 3′-UTR for pMIR-REPORT. **B**, SOSP-9607 cells were transfected with miR-221 mimic, miR-221 inhibitor, scramble oligonucleotide or nothing (blank). And then indicated protein and mRNA were detected. Rows 1 to 7, the expression of PTEN, phospho-Akt (p-Akt), total-Akt (t-Akt), Cyclin D1 (CCND1), p27 and p57 were analyzed by Western blot; row 8, β-actin was used as a loading control; row 9, PTEN mRNA level was measured by RT-PCR; row 10, GAPDH was used as an control. **C**, SOSP-9607 cells were co-transfected with miR-221 mimic or scramble oligonucleotide, and pMIR-REPORT vector consisting of a luciferase gene containing the wild type (PTEN-3′-UTR) or mutated (PTEN-mut-3′-UTR) miR-221 binding sites in its 3′-UTR region by using Lipofectamine 2000 reagent. The cells were also transfected with 50 ng pRL-TK vector as an internal standard. 24 h after transfection, luciferase activity was measured by using a dual luciferase reporter assay (Promega). **D**, similar experiments were performed in MG63 cells as described in panel **C** except that miR-221 mimic was instead by miR-221 inhibitor. The results were presented as relative luciferase activity (firefly Luc/Renilla Luc). All experiments were repeated three times in triplicate. Columns, mean of three independent experiments; bars, SD. **, p<0.01.

Because the miR-221 was one of the most frequently up-regulated miRNAs in many tumors [15]–[19] and has been shown to play an important role in cell survival and cisplatin resistance in both SOSP-9607 and MG63 cells as shown above. We next elucidated the molecular mechanism of miR-221-mediated biological functions. We examined its potential targets by searching the PicTar and miRanda as well as TargetScan database. Among the candidates targets, 3′-UTR of human PTEN contains a putative region (nucleotides 200–206, NM_000314) that matches to the seed sequence of miR-221, which is also conserved in mouse and rat (Figure 5A). Furthermore, to examine whether PTEN is indeed the target of miR-221, SOSP-9607 cells were transfected with 100 nM miR-221 mimic, miR-221 inhibitor, or scramble oligonucleotide. The blank cells were used as a control. Western blot analysis and RT-PCR analyses revealed that PTEN protein but not mRNA was considerably decreased in miR-221 mimic transfected cells. In contrast, knockdown of miR-221 by miR-221 inhibitor increased the protein level of PTEN but not the mRNA level (Figure 5B, row1 and 9). Additionally, the phosphorylation levels of Akt, a major target of PTEN [41] were elevated by ectopic expression of miR-221 and decreased by knockdown of miR-221 (Figure 5B, row2), suggesting that miR-221 targets the PTEN/Akt pathway. The Akt activation pathway is known to be a major cell survival pathway in many cancers [42], [43] and some reports have shown that it was activated in the osteosarcoma cells [44], [45]. Moreover, two downstream genes of pAkt, Cyclin D1 (Figure 5B, row4) and Bcl-2 (Figure 5B, row5) were also analyzed by Western blot. Furthermore, expression of p27, which has previously been described as a miR-221 target [20], [21] and as a Akt pathway downstream gene [31], was reduced after miR-221 over-expression in our model system (Figure 5B, row6). However, expression of p57, which was previously identified as a functional target of miR-221 [46], showed little change with miR-221 overexpression and downexpression (Figure 5B, row7). These results further indicate that the PTEN/Akt pathway is a major target of miR-221 and largely mediates miR-221 function. Next, to investigate whether the repression of PTEN by miR-221 is mediated by direct interaction of miR-221 with PTEN-3′-UTR, we constructed luciferase reporters with wild-type (pMIR-PTEN-3′-UTR) and mutated (pMIR-PTEN-mut-3′-UTR) 3′UTR of PTEN (Figure 5A). Renilla luciferase vectors was used as control. As shown in Figure 5C, luciferase activity of the wild-type PTEN-3′-UTR reporter was significantly suppressed in miR-221 mimic group compared with scramble group (p = 0.002). No statistical significance of luciferase activity of the mutated PTEN-3′-UTR reporter was observed between the miR-221 mimic and scramble group (p = 0.864). In sum, luciferase activity of wild-type PTEN-3′-UTR but not mutated PTEN-3′-UTR reporter was decreased by miR-221 mimic in SOSP-9607 cells (Figure 5C). Moreover, similar experiments were performed in MG 63 cells and similar results were obtained. Luciferase activity of the wild-type, but not mutant, PTEN-3′-UTR reporter was significantly increased in miR-221 inhibitor transfected cells (p = 0.009) (Figure 5D). Together, these data demonstrate that PTEN is a direct target of miR-221 and miR-221 reduces PTEN expression leading to activation of PTEN/Akt pathway.

### Introduction of PTEN cDNA lacking 3′-UTR or PI3K/Akt inhibitor LY294002 abrogates miR-221-induced cisplatin resistance

**Figure 6:**
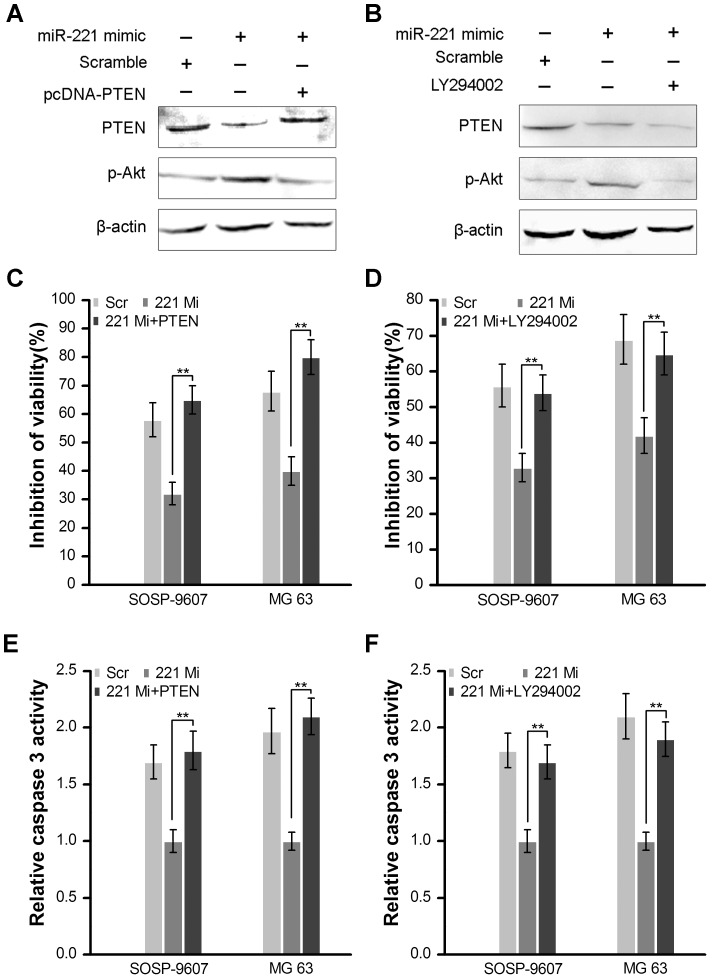
Introduction of PTEN cDNA lacking 3′-UTR or PI3K/Akt inhibitor LY294002 abrogates miR-221-induced cisplatin resistance. **A**, SOSP-9607 cells were transfected with miR-221 mimic (221 Mi), scramble oligonucleotide (Scr) or cotransfected with miR-221 mimic and pcDNA-PTEN. The scramble group was used as a control. The expression of PTEN and phospho-Akt (p-Akt) were analyzed by western blot. β-actin was used as a loading control. **B**, SOSP-9607 cells were transfected with miR-221 mimic, scramble oligonucleotide or cotransfected with miR-221 mimic and LY294002. The scramble group was used as a control. The expression of PTEN and phospho-Akt (p-Akt) were analyzed by western blot. β-actin was used as a loading control. **C**, SOSP-9607 and MG 63 cells were treated as described in panel A. Then the cells were treated with or without cisplatin for 48 h and viability evaluation were examined. Data were presented as the percentage of viability inhibition measured in cells treated without cisplatin. **D**, SOSP-9607 and MG 63 cells were all treated as described in panel B. Then the cells were treated with or without cisplatin for 48 h and viability evaluation were examined. Data were presented as the percentage of viability inhibition measured in cells treated without cisplatin. **E**, both SOSP-9607 and MG 63 cells treated as described in panel A were seeded into 96-well plates and 24 h later were treated with or without cisplatin for 24 h. Caspase3 activity was measured using Caspase-Glo® 3/7 assay kit (Promega) according to the manufacturer's instructions. **F**, both SOSP-9607 and MG 63 cells treated as described in panel B were seeded into 96-well plates and 24 h later were treated with or without cisplatin for 24 h. Caspase3 activity was measured using Caspase-Glo® 3/7 assay kit. Columns, mean of three independent experiments; bars, SD; **, p<0.01, ***, p<0.001.

Because miR-221 directly targets PTEN through interaction between PTEN 3′-UTR and miR-221 (Figure 5), we reasoned that ectopic expression of PTEN by transfection of the cDNA that only contains the open reading frame of PTEN (PTEN -ORF) laking 3′-UTR should escape the regulation by miR-221 and thus attenuate or decrease miR-221 function. To this end, we co-transfected pcDNA3.1-PTEN (only contains the coding region of PTEN and laking 3′-UTR) and miR-221 mimic into SOSP-9607 cells and treated them with or without 10 uM cisplatin. After 48 hours, western blot assay showed that transfection of pcDNA3.1-PTEN efficiently restored PTEN expression and significantly deceased phospho-Akt expression in SOSP-9607 cells transfected with miR-221 mimic (Figure 6A). And MTT assay showed restoring expression of PTEN and decreasing expression of phospho-Akt by co-transfecting pcDNA3.1-PTEN recovered the cisplatin sensitivity in both miR-221 transfected SOSP-9607 and MG 63 cells (Figure 6C). Moreover, caspase3 activity induced by cisplatin were also significantly increased by ectopic expression of PTEN in miR-221 transfected SOSP-9607 and MG 63 cells (Figure 6E). Furthermore, we reasoned that inhibition of Akt should override miR-221-induced cisplatin resistance. To test this hypothesis, miR-221 transfected SOSP-9607 cells were treated with a specific PI3K/Akt inhibitor LY294002 (10 uM), in combination with or without 10 uM cisplatin. The cells transfected with scramble oligonucleotides were used as control. As expected, 48 h after transfection, LY294002 significantly abrogated miR-221-activated Akt (Figure 6B) and significantly inhibited miR-221-induced cisplatin resistance in both SOSP-9607 and MG63 cells (Figure 6D). Moreover, LY294002 also inhibited the caspase3 activity of both SOSP-9607 and MG63 cells (Figure 6F). These results further indicate that the PTEN/Akt pathway is a major target of miR-221 and largely mediates miR-221 cisplatin resistance function.

### Inverse correlation of expression of miR-221 and PTEN in human osteosarcoma samples

**Figure 7:**
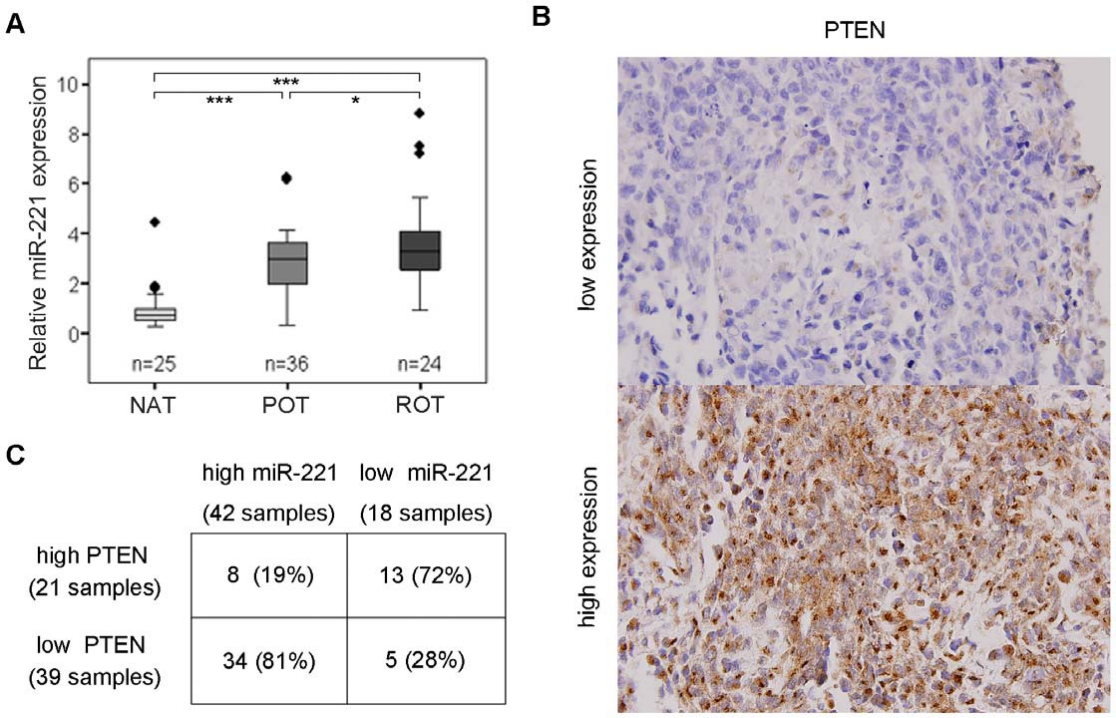
Inverse correlation of expression of miR-221 and PTEN in human osteosarcoma samples. **A**, expression of miR-221 was analyzed by qRT-PCR in normal adjacent tissues (NAT), primary osteosarcoma tissues (POT) and recurrent osteosarcoma tissues (ROT). U24 was used as internal control. The average expression values of adjacent normal tissues is set at 1. Horizontal bars in the box represent median. *, p<0.05, ***, p<0.001. **B**, expression of PTEN was analyzed in osteosarcoma tissues with immunohistochemical staining. **C**, Chisquare test analysis of miR-221 and PTEN expression. The inverse correlation is significant (p<0.001).

Having demonstrated PTEN as a major target of miR-221 in osteosarcoma cell lines, we next investigated the miR-221 expression and the correlation between miR-221 and PTEN expression in osteosarcoma tissues. As initial step, we examined miR-221 expression by real-time quantitative PCR in 25 normal adjacent tissues and 60 osteosarcoma tissues (including 36 primary osteosarcoma tissues and 24 recurrent osteosarcoma tissues). Real-time quantitative PCR analysis showed miR-221 expression levels in recurrent and primary osteosarcoma tissues were significantly higher than that in normal adjacent tissues (each p<0.001). In addition, we also observed that miR-221 is significantly increased in these recurrent tissues when compared with primary tissues (p<0.05) (Figure 7A). Moreover, we examined PTEN expression in these 60 osteosarcoma specimens with immunohistochemical staining. Representative images of PTEN were shown in Figure 7B. Of the 42 osteosarcoma cases with elevated miR-221, 34 (81%) had low levels of PTEN (p<0.001). 13 of 18 (72%) cases with down regulated miR-221 presented high levels of PTEN (Figure 7C). These findings suggest that miR-221 regulation of PTEN *in vivo* and that miR-221 plays an important role in chemoresistance.

## Discussion

Increasing evidences indicate that miRNAs may exert functions as oncogenes or tumor suppressors in human cancers depending on the role of the their targets [6]–[13]. Precisely, miRNAs may be considered as promising molecular targets for therapy of human cancers. miR-221 is one of the most commonly and highly upregulated miRNA in cancer and thus, one of the most studied [15]–[21]. Le Sage *et al.* showed that upregulated expression of miR-221 promotes glioblastomas cell proliferation by inhibiting the expression of p27 [20]. Galardi *et al.* reported upregulated miR-221 expression enhanced the proliferation potential of human prostate carcinoma cell lines by targeting p27 [21]. Garofalo *et al.* found that miR-221 regulated TRAIL resistance and enhanced tumorigenicity through PTEN and TIMP3 downregulation [18]. Chunzhi *et al.* demonstrated miR-221 and miR-222 could regulate gastric carcinoma cell proliferation and radioresistance by targeting PTEN [16]. Accumulating evidences demonstrated that miR-221 was an “oncomir” and played a key role in human cancers. However, little is known regarding the impact of miR-221 on PTEN in human osteosarcoma and chemotherapy resistant. In this study, we focused on the function of miR-221 in human osteosarcoma cells. As initial step, we evaluated that the expression of miR-221 was significantly increased in these osteosarcoma cell lines when compared with hFOB1.19 osteoblasts cells (Figure 1A). These results are also consistent with previous studies that have shown increased expression of miR-221 other human cancers [15]–[21], suggesting that increased miR-221 levels are not tumor-type specific. For investigating the function of miR-221, we used chemically synthesized oligonucleotides mimic or inhibit the function of miR-221. Experiments showed that these oligonucleotides could be efficiently transfected into SOSP-9607 cells (Figure 1B, top) and MG63 cells (Figure 1B, bottom) and could significantly increased or decreased miR-221 expression levels (Figure 1C and D). In the present study, we have shown for the first time that overexpression of miR-221 regulate osteosarcoma cell viability, apoptosis, cell cycle progression and cisplatin resistance. Our data suggest that downregulation of PTEN expression and enhanced Akt phosphorylation (p-Akt) are important mediators of these cellular processes. As p-Akt impacts cell proliferation, cell transit from the G0/G1 to the S phase, apoptosis and cell cisplatin resistance, downregulation of miR-221 expression have important biologic effects on the malignant phenotype of both SOSP-9607 and MG63 cells. These results identify down-regulated miR-221 as a potential therapeutic approach for human osteosarcoma cells via PI3K/Akt pathway by targeting PTEN.

The PI3K/Akt pathway is well known to be a major cell survival pathway and activation of the PI3K/AKT pathway enhances resistance to apoptosis [22]–[25]. Cenni *et al.* showed that inhibition of the Akt pathway could make multidrug resistant human osteosarcoma cells more sensitive to Apo2 Ligand/TRAIL-induced apoptosis [28]. Inoue *et al.* demonstrated that alendronate inhibited the cell survival stimulated by the PI3K/Akt pathway and thus caused apoptosis of osteosarcoma cells [29]. These results suggested that the PI3K/Akt pathway was also activated in the osteosarcoma cell line. PTEN functions as a tumor suppressor gene, specifically by negatively regulating the PI3K/Akt signaling pathway [35]. PTEN could antagonize PI3K activity by dephosphorylating PIP3 and thereby negatively regulate the activity of Akt pathway [31], [34]. However, little is known regarding the impact of miR-221 on PTEN expression in osteosarcoma cells. In the present study, we predicted binding sites for miR-221 in the PTEN 3′-UTR by bioinformatics analysis (Figure 5A). Western blot assay showed that increased expression of the miR-221 might impact on PTEN expression (Figure 5B). Indeed, we demonstrated that PTEN was a target gene of miR-221 by luciferase reporter assay (Figure 5C and D). Moreover, miR-221 targets PTEN leading to activation of the Akt pathway. In our study, we found that knockdown of miR-221 in SOSP-96607 cells resulted in downregulation of p-Akt expression (Figure 5B, row2), affecting the expression of several Akt-regulated proteins including cyclin D1 (Figure 5B, row4) and Bcl-2 (Figure 5B, row5) and p27 (Figure 5B, row6). For this reason, miR-221 modulates a variety of biological functions in the SOSP-9607 and MG 63 cells, including cell proliferation (Figure 2A and B), cell cycle (Figure 2C) and apoptosis (Figure 3).

Cisplatin is still one of the most common used agents in chemotherapy due to the therapeutic advantages, such as high efficiency, mild side effects and easy administration. However, resistance to this drug is also often observed, and therefore enhancing the sensitization of cancer cells to cisplatin-induced apoptosis has became an important strategy for chemotherapy. Wang *et al.* found that down-regulation of P28GANK gene expression could sensitize osteosarcoma cells to cisplatin by down-regulation of the MDR-1 and Bcl-2 and up-regulation of Bax gene expression [36]. Pasello *et al.* confirmed that overcoming glutathione S-transferase P1-related cisplatin resistance in osteosarcoma [37]. Dai *et al.* found TSSC3 knockdown increased Saos2 cell growth and decreased apoptosis *in vitro* and *in vivo* and reduced sensitivity of the cells to chemotherapy [38]. Huang *et al.* found HMGB1 promoted drug resistance in osteosarcoma [39], [40]. MicroRNAs are also found as novel modulators of the cisplatin response. Sun *et al.* found that downexpression of miR-200b was associated with chemotherapeutic resistance in tongue squamous cell carcinoma (TSCC) cell lines by targeting BMI1 [41]. It was reported that miR-200bc/429 cluster could play a role in the development of MDR in both gastric and lung cancer cell lines via targeting BCL2 and XIAP [42]. Galluzzi *et al.* reported that miR-181a and miR-630 enhanced and reduced CDDP-triggered cell death in A549 cells, respectively [43]. However, the precise roles of miRNAs in chemotherapeutic resistance in osteosarcoma cells have not been well elucidated. In the present study, we found for the first time that overexpression of miR-221 markedly inhibited cisplatin-induced cell cytotoxicity in both SOSP-9607 cells and MG63 cells while downexpression of miR-221 markedly induced cisplatin-induced cell cytotoxicity in these cells (Figure 4A–D). Moreover, measurement of caspase3 activities confirmed that silencing miR-221 expression could enhance cisplatin-induced apoptosis, further explaining clearly the signaling pathway of cisplatin-induced apoptosis in osteosarcoma (Figure 4E and F). Furthermore, we observed that inhibition of Akt with LY294002 or transfection of pcDNA-PTEN lacking 3′-UTR overrided miR-221-induced cell resistance to cisplatin (Figure 6C and D). These results indicate that miR-221 plays an important role in cisplatin resistance by targeting PTEN/Akt pathway. Bcl-2, as a downstream gene of p-Akt, was also regulated by miR-221 in cisplatin treatment cells (Figure 5B, row5), corroborating the results of Wang *et al.* [36] and Zhu *et al.* [42]. Several studies suggest that CCND1 is an important regulator of cell cycle progression at G1/S transition and its overexpression is implicated as driving feature in various types of cancer such as mantle cell lymphoma (MCL) [44], non-small cell lung cancer [45], prostate cancer cells [46], gliomas [47], osteosarcoma [48], [49] and so on. Increased cyclin D1 translation is mediated by the PI3K/Akt cascade [50]. Cyclin D1 overexpression is frequently associated with human malignancy whereas cyclin D1 downexpression enhance G1 phase arrest. p27 is a negative regulator of the G1 phase of the cell cycle and its overexpresssion results in cell cycle arrest in G1 phase. It has been reported that p27 could be regulated by PI3K/Akt pathway [31]. And recently, p27 was confirmed as a direct target of miR-221 [20], [21]. In this study, the expression of CCND1 and p27 protein was also inceased by miR-221 mimic and deceased by miR-221 inhibitor (Figure 5B, row4). The expression changes of these two genes could contribute to G1/S transition in both SOSP-9607 and MG63 cells (Figure 2C). Thus, our findings are consistent with an emerging body of literature. Interestingly, a recent report demonstrated that BMI-1 functioned as an oncogene in osteosarcoma and overexpression of BMI-1 promoted cell growth and resistance to cisplatin treatment in osteosarcoma through PI3K/AKT pathway [51]. Ours findings were also consistent with theirs.

Unlike siRNAs which silence the expression of a single gene, miRNAs mainly silence the expression of multiple genes simultaneously. It is estimated that an average miRNA has more than 100 targets [52], and miRNAs have the potential to regulate at least 20%–30% of all human genes [53]. We reviewed experimentally identified miR-221 targets or upstream genes and pathways. Among them, there are several genes and pathways mediate osteosarcoma tumorigenicity such as p57 [54], [55], NF-κb [56], [57], p27 [21], [58], Notch pathway [59] and so on. Our work identified a role for PTEN in miRNA-221-induced biology and confirmed p27 but not p57 could be regulated by miR-221, these results indicate that miR-221 induces cisplatin resistance directly through at least p27 and PTEN pathway, it remains possible other factors might be partially involved. miR-222, which is also clustered on the X chromosome, and may possibly be transcribed from a common precursor, has a coordinate functional role with miR-221 [15]–[21]. What is the specific function of miR-222 in osteosarcoma needs further experiments. Thus, in general, the discovery of miRNA and their functions, is introducing a new dimension to our existing knowledge of signaling molecules and pathways that remains to be explored and exploited for more precise therapeutic targeting. Further investigations are required for characterization of miR-221 and other miRNAs as prognostic and/or diagnostic markers in human osteosarcoma.

In summary, our study demonstrated that miR-221 induced cell survival and cisplatin resistance at least through PI3K/Akt pathway in human osteosarcoma by direct targeting PTEN. We also provided direct evidence using miR-221 inhibitor as therapeutic approaches for osteosarcoma. The roles of miR-221 and other miRNAs in osteosarcoma cells need to be further investigated.

## Materials and Methods

### Ethics statement

All research involving human tissue samples have been approved by the Ethics Review Committee of Fourth Military Medical University, Xi'an, Shaanxi, China (approval ID:2012089) and written informed consent was obtained from all participating patients.

### Cell lines and human osteosarcoma samples

Human osteosarcoma cells SOSP-9607 and SOSP-9901 were established and reserved in our laboratory as described previously [60]. Cells were grown in RPMI 1640 medium (HyClone, USA) supplemented with 10% fetal bovine serum (FBS) serum, 2 mM glut amine, 100 U/ml penicillin, and 100 mg/ml streptomycin at 37°C in 5%CO_2_ and 95% atmosphere. MG63, Saos-2, U2OS, hFOB1.19 cells were obtained from the American Type Culture Collection (ATCC, Rockville, MD, USA), cells were maintained in the same conditions, except that DMEM medium (for MG 63, HyClone, USA), McCoy's 5A medium (for Saos-2 and U2OS, Gibco, USA) and phenol red-free DMEM/F12 medium (for hFOB1.19, Invitrogen, USA) were used.

All 36 primary and 24 recurrent human osteosarcoma tissues and 25 normal adjacent tissues were obtained from patients who underwent surgery at Orthopedics Oncology Institute of Chinese PLA of Tangdu Hospital. Immediately after surgery, tissues were snap-frozen and stored in liquid nitrogen until further use.

### RNA oligonucleotides, plasmids and transfection

The FAM modified 2′-OMe-oligonucleotides were chemically synthesized and purified by high-performance liquid chromatography by GenePharma Co. Ltd. (Shanghai, China). The 2′-O-me-miR-221 mimic was composed of RNA duplexes with the following sequence: 5′-AGCUACAUUGUCUGCUGGGUUUC/AACCCAGCAGACAAUGUAGCUUU-3′; The sequences of 2′-O-me-miR-221 inhibitor and 2′-O-me-scramble oligonucleotide were as follows: 5′-GAAACCCAGCAGACAAUGUAGCU-3′; and 5′-CAGUACUUUUGUGUAGUACAA-3′. All the oligonucleotides were 2′-OMe modified. There was a FAM fluorescent label in 5′ oligonucleotide structure in every oligonucleotide. Wild-type PTEN lacking the 3′UTR region was constructed into the pcDNA vector (pcDNA3.1-PTEN) by Genesil Biotechnology Co. Ltd. (Wuhan, China). When cells were grown to 70–80% confluence, transfection was performed using the Lipofectamine™ 2000 transfection reagent (Invitrogen, USA) according to the manufacturer's instructions. At 4 h after infection, the medium was replaced with fresh medium containing 10% fetal bovine serum.

### Quantitative real-time PCR and RT-PCR

Total RNA containing miRNA and mRNA was extracted by using Trizol reagent (Invitrogen, USA) according to the manufacturer's instructions. RNA was resuspended in DEPC-treated H_2_O.

For evaluating the miR-221 expressing levels, quantification using the TaqMan microRNA assays was performed using two-step RT-PCR according to the manufacturer's instructions. In the reverse transcription (RT) step, cDNA was reverse transcribed from total RNA sample using specific miR-221 primers from the Taqman® MicroRNA Assays kit (Applied Biosystems, Product ID:000524) and reagents from the TaqMan® MicroRNA Reverse Transcription Kit (Applied Biosystems, Part Number:4366596). In the Polymerase Chain Reaction (PCR) step, PCR products were amplified from cDNA samples using the Taqman® MicroRNA Assays kit together with the TaqMan® Universal PCR Master Mix (Applied Biosystems, Part Number:4304449). The real-time PCR results were normalized against an internal control U24 small nucleolar RNA (RNU24) and the relative expression levels were evaluated using the 2^−ΔΔCt^ method and then expressed as fold changes. For tissues, the expression of miR-221 was determined by comparing the miR-221/U24 ratio between normal adjacent tissues and osteosarcoma tissues. The average ratio of miR-221/U24 in normal adjacent tissues was considered as 1.0 and high value for overexpression of miR-221 is ≥2-fold.

For reverse transcription PCR, 1 ug of total RNA was used for reverse transcription with iScript cDNA Synthesis Kit (Bio-Rad, USA) according to the manufacturer's instructions and PCR were performed using the MyCycler Thermal Cycler 580BR-3001 system (Bio-Rad, USA) and gene-specific primers. The sequences of primers were as follows: PTEN, F: 5′-GGTTCACATCCTACCCCTTTG-3′, R: 5′-TTCTGAGCATTCCCTCCATTC-3′; GAPDH, F: 5′-TGGGTGTGAACCATGAGAAGT-3′, R: 5′-TGAGTCCTTCCACGATACCAA-3′. The expression level of GAPDH was used as a control. The PCR conditions were 25–30 cycles at 95°C for 30 s, 56°C for 30 s, and 72°C for 1 min. The PCR products were separated on a 2% agarose gel. All reactions were performed at least in triplicate.

### Cell viability assay

Cell viability was examined with 3-(4,5-dimethylthiazol-2-yl)-2,5-diphenyltetrazolium bromide (MTT) assay. For cells proliferation viability assay, cells were seeded into 96 well plates at a density of 1500 cells/well containing 100 ul of culture medium and cultured overnight. Cells were transfected with 100 nM miR-221 mimic, miR-221 inhibitor or scramble oligonucleotide. Cells transfected nothing (blank) were used as control. Fresh complete medium was changed for cells at 24 h post-transfection except for those cells to be tested for viability at this time point. Every 24 h interval, 20 µl of 5 mg/mL MTT (Dimethyl thiazolyl diphenyl tetrazolium, Sigma) was added into each corresponding test well, and incubated for 4 h in humidified incubator. The supernatant was then discarded, and 200 µL of DMSO (dimethyl sulfoxide) was added to each well to dissolve the formazan. Optical density (OD) was evaluated by measuring the absorbance, with a test wavelength of 490 nm and a reference wavelength of 630 nm. Wells without cells (DMSO alone) were used as a control to normalize the results. The final optical (OD) density was calculated according to the following formulae: final optical density = optical density of each group - optical density of DMSO group. Each test was performed daily for five consecutive days and repeated in five wells. The experiments were repeated three times independently and the results were given as means ± SD.

For cisplatin-induced cytotoxicity assay, cells were transfected with 100 nM miR-221 mimic, miR-221 inhibitor or scramble oligonucleotides. The blank (transfected nothing) group was used as control. 24 h after transfection, cells were seeded into 96 well plates at a density of 5000 cells/well containing 100 ul of culture medium and cultured overnight. Then cells were teated with cisplatin or not. Cell viability assay was examined with MTT as described above. % viability survival was calculated according to the following formulae: % viability survival = final OD value of cells with cisplatin/final OD value of corresponding cells without cisplatin×100%. And then inhibition of viability (%) = 1 - % viability survival. For combination experiments with miR-221 mimic and pcDNA-PTEN or LY294002, cells were treated and seeded as described above, plus cotransfection with pcDNA-PTEN (0.2 ug) or incubation LY294002 (10 µM), combined with miR-221 mimic before the incubation with or without cisplatin in 96-well plates. Then inhibition of viability of cells was examined using MTT. The experiments were repeated three times independently and the results were given as means ± SD.

### Cell cycle analysis and apoptosis assay by flow cytometry

24 h before transfection, SOSP-9607 and MG63 cells were seeded into 6-well plates at a density of 1×10^5^ cells/well. The following day, cells were transfected with 100 nM miR-221 mimic, miR-221 inhibitor or scramble oligonucleotides. The blank (transfected nothing) group was used as control. 48 h after transfection, cells were analyzed by flow cytometry. For cell cycle analysis, the transfected SOSP-9607 and MG 63 cells in the log phase of growth were stained with the DNA-binding dye propidium iodide (50 µg/ml) and RNase (1.0 mg/ml) for 30 min at 37°C in the dark and examined with a fluorescence-activated cell-sorting (FACS) flow cytometer (BD Biosciences, USA), and DNA histograms were analyzed with modified software. For apoptosis assay, 1×10^5^ treated cells were incubated with annexin V/propidium iodide for 15 min at room temperature in the dark. The cells were then analyzed by flow cytometry using fluorescence-activated cell-sorting (FACS) flow cytometer (BD Biosciences, USA). Each test was repeated in triplicate.

### Target prediction

Bioinformatics analysis was performed by using these specific programs: Pictar (http://pictar.mdc-berlin.de/), miRanda (http://www.microrna.org) and Targetscan (http://www.targetscan.org/).

### Western blot analysis

Transfected SOSP-9607 cells were washed with pre-chilled PBS and then were subjected to lysis in a RIPA buffer with 0.5% sodium dodecyl sulfate (SDS) in the presence of 3% proteinase inhibitor cocktail (Sigma, USA) on ice. 30 min later, the lysate was centrifuged at 12,000 rpm for 20 min, and the supernatant was collected as total proteins for experiments. Equal amounts of protein lysates (40 µg each) was separated by 4–20% SDS-PAGE and then electrotransferred to nitrocellulose membranes(Invitrogen, USA). The membranes were blocked with TBST containing 5% non-fat dry milk for 1 h and incubated with primary antibody against PTEN (abcam, China), phospho-Akt (Cell Signaling Technology, Canada), total-AKT (Cell Signaling Technology, Canada), cyclin D1 (abcam, China), Bcl-2 (abcam, China), p27 (Santa Cruz, USA), p57 (abcam, China), β-Actin (Santa Cruz, USA) overnight at 4°C. After washed 3 times with TBST, the membranes were incubated with horseradish peroxidase conjugated anti-mouse or rabbit secondary antibody (Santa Cruz, USA) for 2 h. Then washed 3 times with TBST again, the specific protein was detected by chemiluminescence using Pierce ECL Western Blotting Substrate (Thermo Fisher Scientific, USA). β-Actin was used as a control. The optical density of protein fragments was quantified by Quantity One software (Bio-Rad, USA).

### Luciferase reporter assay

A 229-bp fragment of the human PTEN 3′UTR containing the miR-221 binding side was amplified by PCR from SOSP-9607 genomic DNA using primers: PTEN-3′UTR-F, 5′-CGGACTAGTCTATACATCCACAGGGTT-3′ and PTEN-3′UTR-R, 5′-CCCAAGCTTTCTTTAGCCACTTCAGTT-3′ and was cloned into downstream of the firefly luciferase gene present in the pMIR-REPORT vector (Ambion, USA) between *Hind*III and *Spe*I sites to develop the pMIR-PTEN-3′-UTR luciferase vector. Seed sequences of miR221-binding sites in PTEN-3′-UTR fragment were mutated using the QuikChange Mutagenesis Kit (Stratagene, USA). The mutate PTEN-3′-UTR fragment was cloned into pMIR-REPORT vector to develop the pMIR-PTEN-mut-3′-UTR vector. For the luciferase assay in SOSP-9607 cells, cells at the density of 1.2×10^5^ per well in 24-well plates were cotransfected with 0.8 ug pMIR-REPORT luciferase reporters with 3′-UTR or mut-3′-UTR of PTEN, 100 nM miR-221 mimic or scramble oligonucleotide using Lipofectamine 2000 reagent. The cells were also transfected with 50 ng pRL-TK vector as an internal standard. For the luciferase assay in MG 63 cells, cells were treated in the same conditions, except that miR-221 mimic was instead by miR-221 inhibitor. Twenty-four hours later, luciferase activity was measured by using a dual luciferase reporter assay (Promega, USA) on a Berthold AutoLumat LB9507 rack luminometer. The results were expressed as relative luciferase activity (firefly Luc/Renilla Luc). All experiments were repeated three times in triplicate.

### Caspase3 activity assay

For detection of caspase3 activity, cells were cultured in 96-well plates and treated with the agents indicated in the figure legends and analyzed using Caspase-Glo® 3/7 assay kit (Promega, USA) according to the manufacturer's instructions.

### Immunohistochemistry

The dilution of PTEN antibody used for immunohistochemistry were 1∶100. Immunohistochemistry were performed as described previously [61]. The final scores of PTEN expression were calculated as described previously [62] and classified as follows: 0–4, low; 5–9, high.

### Statistical analysis

All values in the paper were expressed as the mean ± SD, all error bars represent the standard deviation of the mean. Student's t test and one-way analysis of variance was used to determine significance. All statistical tests were two-sided. *p*<0.05 was considered statistically significant. Statistical analyses were performed using SPSS 13.0 statistics software (SPSS Inc, USA).
