# Correction: MicroRNA-221 Induces Cell Survival and Cisplatin Resistance through PI3K/Akt Pathway in Human Osteosarcoma

**DOI:** 10.1371/annotation/f47e2af6-da90-40ee-871c-f9ed6f58a48c

**Published:** 2013-05-28

**Authors:** Guangyi Zhao, Chengkui Cai, Tongtao Yang, Xiuchun Qiu, Bo Liao, Wei Li, Zhenwei Ji, Jian Zhao, Haien Zhao, Mingjun Guo, Qiong Ma, Chun Xiao, Qingyu Fan, Baoan Ma

In the Figure 3 of our article, different gates were set for SOSP-9607 or MG63 cells. A reader noted that the same gates should be set in SOSP-9607 or MG63 cells for flow cytometry.

In order to address a reader's concerns and in line with the editor's recommendation, we are providing a revised Figure 3. In the new Figure 3, the same gates were reset for SOSP-9607 or MG63 cells from the original data. As a result, the histograms in panel C and D have changed slightly.

The revised Figure 3 can be viewed here: 

**Figure:**
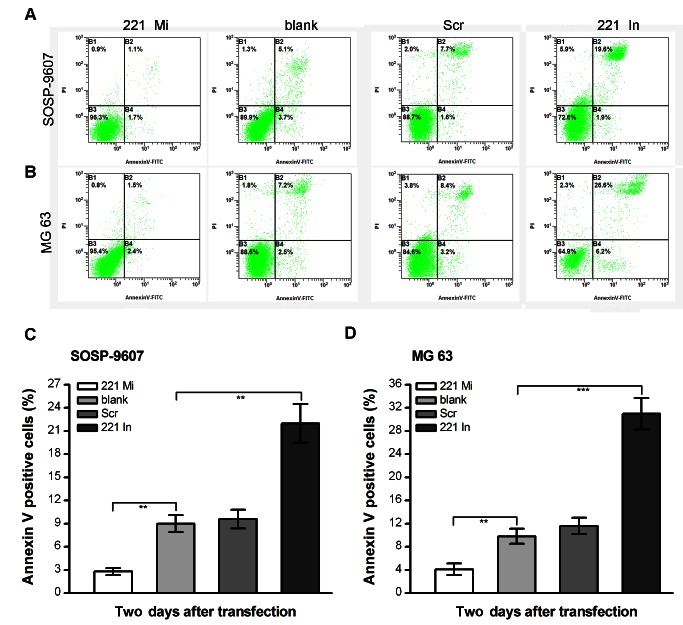


The modifications to this figure do not affect the results and conclusions reported in the article. 

